# The effect of hip abduction on the EMG activity of vastus medialis obliquus, vastus lateralis longus and vastus lateralis obliquus in healthy subjects

**DOI:** 10.1186/1743-0003-3-13

**Published:** 2006-07-03

**Authors:** Débora Bevilaqua-Grossi, Vanessa Monteiro-Pedro, Rodrigo Antunes de Vasconcelos, Juliano Coelho Arakaki, Fausto Bérzin

**Affiliations:** 1 Department of Biomechanics, Medicine and Rehabilitation of Locomotor Apparatus, Ribeirão Preto School of Medicine, University of São Paulo (FMRP-USP), Ribeirão Preto, SP, Brazil; 2 Department of Physical Therapy, Federal University of São Carlos (UFSCar), SP, Brazil; 3 Department of Physical Therapy, University for Development of Pantanal State and Region (UNIDERP), Campo Grande, MS, Brazil; 4 Department of Morphology, Piracicaba School of Dentistry, State University of Campinas (FOP-UNICAMP), Piracicaba, SP, Brazil

## Abstract

**Study design:**

Controlled laboratory study.

**Objectives:**

The purposes of this paper were to investigate (d) whether vastus medialis obliquus (VMO), vastus lateralis longus (VLL) and vastus lateralis obliquus (VLO) EMG activity can be influenced by hip abduction performed by healthy subjects.

**Background:**

Some clinicians contraindicate hip abduction for patellofemoral patients (with) based on the premise that hip abduction could facilitate the VLL muscle activation leading to a VLL and VMO imbalance

**Methods and measures:**

Twenty-one clinically healthy subjects were involved in the study, 10 women and 11 men (aged X = 23.3 ± 2.9). The EMG signals were collected using a computerized EMG VIKING II, with 8 channels and three pairs of surface electrodes. EMG activity was obtained from MVIC knee extension at 90° of flexion in a seated position and MVIC hip abduction at 0° and 30° with patients in side-lying position with the knee in full extension. The data were normalized in the MVIC knee extension at 50° of flexion in a seated position, and were submitted to ANOVA test with subsequent application of the Bonferroni multiple comparisons analysis test. The level of significance was defined as p ≤ 0.05.

**Results:**

The VLO muscle demonstrated a similar pattern to the VMO muscle showing higher EMG activity in MVIC knee extension at 90° of flexion compared with MVIC hip abduction at 0° and 30° of abduction for male (p < 0.0007) and MVIC hip abduction at 0° of abduction for female subjects (p < 0.02196). There were no statistically significant differences in the VLL EMG activity among the three sets of exercises tested.

**Conclusion:**

The results showed that no selective EMG activation was observed when comparison was made between the VMO, VLL and VLO muscles while performing MVIC hip abduction at 0° and 30° of abduction and MVIC knee extension at 90° of flexion in both male and female subjects. Our findings demonstrate that hip abduction do not facilitated VLL and VLO activity in relation to the VMO, however, this study included only healthy subjects performing maximum voluntary isometric contraction contractions, therefore much remains to be discovered by future research

## Introduction

Patellofemoral pain syndrome (PFPS) presents one of the most perplexing pathologic conditions in orthopedic and sports medicine clinics, as well as in rehabilitation departments, and it was referred to by Dye [1] as the "black hole of orthopedics" because of the lack of clarity regarding the etiological factors that contribute to dysfunction or to specific treatment protocols and the causative mechanisms remain imprecisely defined [2].

Dysfunction of the quadriceps muscle has been hypothesized as a cause of patellofemoral pain syndrome (PFPS) with great emphasis placed on the role of VMO and VL muscular imbalance [3,4]. Quadriceps dysfunction in PFPS patients has been assessed in various ways including decreased magnitude of the electromyographic (EMG) activity of the quadriceps [5,6], diminished EMG activity of the VMO in relation to that of the VL [7-9], and the delayed onset of VMO activation in relation to the VL [10-12] caused by the inhibition of pain, effusion and atrophies[13]. Improved control of patella tracking is necessary for symptomatic relief [14] and the recovery of quadriceps function is essential to the resolution of the problem [15]. Consequently, there have been numerous studies that have sought to identify exercises to selectively recruit the VMO in an effort to retrain this muscle [7,16-18]. However, Mirzabeigi et al. [19] suggested that the VMO muscle cannot be significantly isolated during nine sets of different exercises.

**Figure 1:**
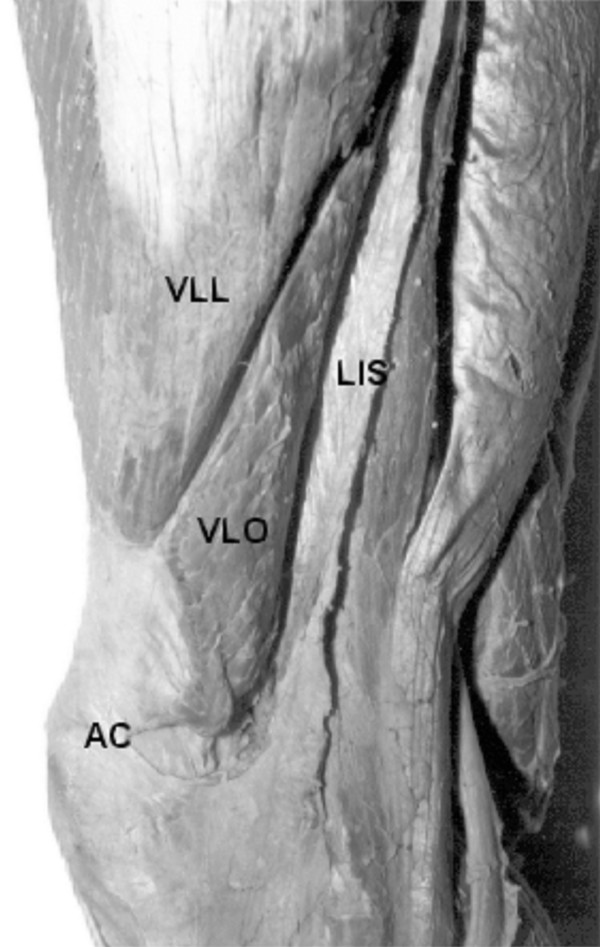
Lateral view of the right thigh showing the origin of the obliquus portion of the vastus lateralis muscle (vastus lateralis obliquus – **VLO**) in the lateral intermuscular septum (**LIS**) and its insertion in the superior -lateral border of the patella (**P**). **VLL **– vastus lateralis longus. Bevilaqua-Grossi et al. (2004) ^46^.

Since fibers of the VMO attach to the adductor magnus muscle, it has been hypothesized that activation of the VMO may be enhanced by combining active knee extension with volitional hip adduction [4,16,20]. Research in clinical studies on the treatment of PFPS has reported that VL activity may also be enhanced by combining knee extension exercises with hip abduction [21,22]. Fulkerson [23] noted retinacular tenderness in patients with patellofemoral pain, including some who demonstrated histological changes within the perineural tissues of the lateral retinaculum on examination of surgical biopsy specimens. Bevilaqua-Grossi et al. [24] in their anatomical study dissected the thighs of 32 human cadavers and showed that the distal fibers of the Vastus Lateralis Obliquus (VLO) were interdigitated with the lateral retinaculum and the iliotibial tract in all specimens, were subsequently joined in a common tendon with the VLL on the superolateral border of the patella (Figure 1) and that tightness of lateral retinaculum could potentially alter the tracking of the patella in the trochlear groove. Based on this anatomic correlation, Hip abduction exercises are often contraindicated by physical therapists because some group of patient with patellofemoral problems may have tight lateral structures and hip abduction exercises would enhance VMO and VL muscular imbalance [21].

Considering that previous few studies have investigated the EMG relationship VMO and VLO with other structures of the lateral compartment (lateral retinaculum and iliotibial tract) and hip abduction strengthening exercises, the aim of this paper was to analyze the EMG activity of the VMO, VLL and VLO muscles and verify whether any difference in activity between these portions occurred during MVIC: 1) knee extension at 90° of flexion, 2) hip abduction at 0° of abduction and 3) hip abduction at 30° of abduction. The data reported in this paper should be useful in future functional studies aimed at a clearer understanding of rehabilitation protocols in PFPS patients.

## Methods

### Subjects

Twenty-one healthy volunteers (11 males and 10 females), aged from 19 to 28 (X = 23.3 ± 2.9), participated in this study. They were recruited from Piracicaba Methodist University and all reported no history of orthopedic disorders, surgical procedures, knee pain or other major musculoskeletal injuries. Prior to participation, all subjects read, accepted and signed a consent form that was approved by the Human Research Ethics Committee at the State University of Campinas.

### Instrumentation

Silver/silver chloride surface electrodes placed in a bipolar configuration, with a 10 mm contact area and an inter-electrode distance of 2 cm, were used to assess the level of electromyographic activity of the VMO, VLL and VLO muscles. Before the electrode placement the sites were prepared by shaving, abrading and cleaning with isopropyl alcohol to reduce the surface impedance to less than 5 and 10 kΩ for men and women, respectively. A quadriceps line was drawn from the anterior superior iliac spine to the center of the patella for the quadriceps portion placement [24]. A surface electrode for the VMO was placed with a medial inclination of 55° from the quadriceps line [25,26]. The VLL electrode was placed 15 cm from the superior edge of patella at a lateral inclination of 13.6° and the VLO shows its superficial part around 2.2 cm of the lateralis epicondyle with a superficial length of around 8.9 cm with a 50.4° of lateral inclination[27]. A ground electrode was placed over the tibial tubercle of the tested lower limb. The same investigator performed all electrode placements.

A calibrated Viking II with eight channels (Nicolet Biomedical Instruments) and a computer were used to collect all EMG data (CMRR of 110 dB, sampling 1000 Hz, gain 200, band pass filter of 10 to 1000 Hz). All signals were viewed on a display screen prior to collection to ensure that there were no visible artifacts.

### Procedures

Following EMG preparation, the subjects were instructed to perform 3 repetitions of the following three sets of exercises: 1) MVIC knee extension at 90° of flexion in a seated position; 2) MVIC hip abduction at 0° of abduction, with patients in side-lying position with the knee in full extension; 3) MVIC hip abduction at 30° of abduction, with patients in side-lying position with the knee in full extension

**Figure 2:**
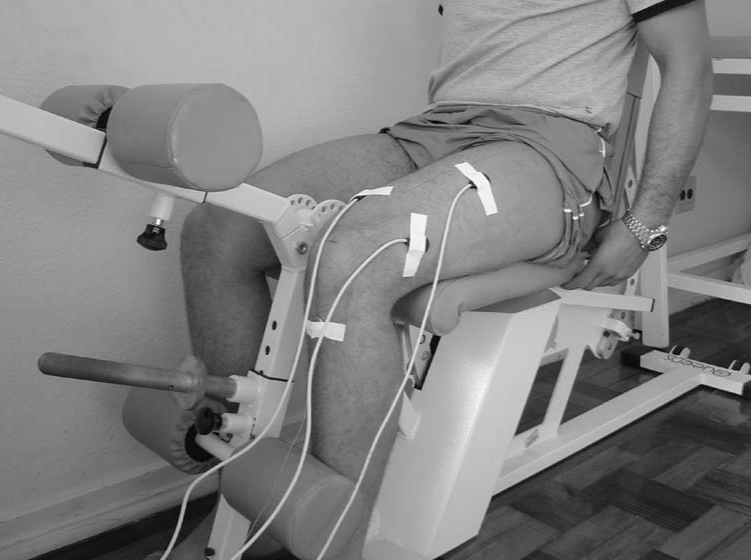
Maximum voluntary isometric contraction at 90 degrees of knee extension.

**Figure 3:**
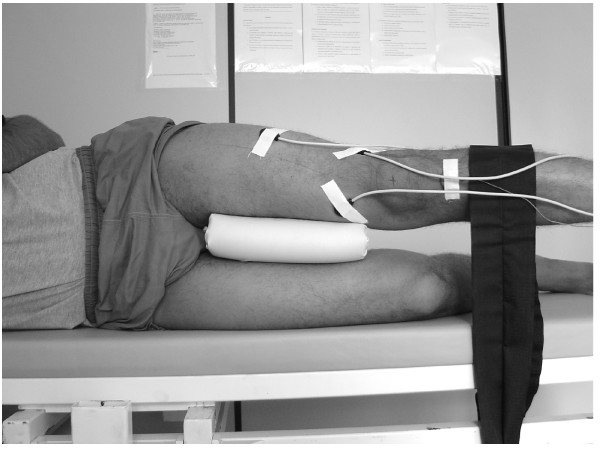
Maximum voluntary isometric contraction of hip abduction in neutral in side-lying position. The subjects were instructed to maintain an isometric quadriceps contraction at full knee extension while performing the task.

**Figure 4:**
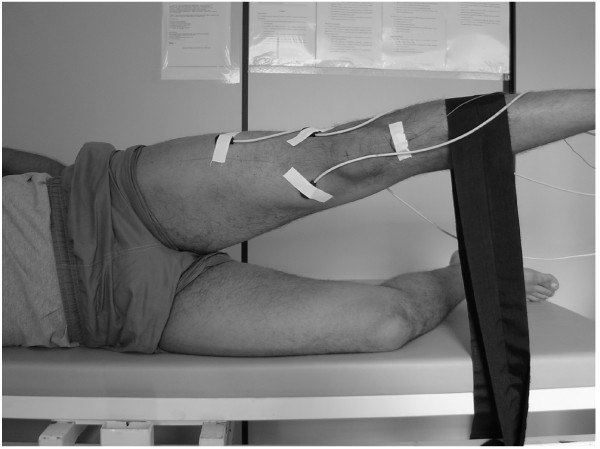
Maximum voluntary isometric contraction at 30 degrees of hip abduction in side-lying position. The subjects were instructed to maintain an isometric quadriceps contraction at full knee extension while performing the task.

During the MVIC knee extension test, the subjects were positioned seated in a leg extension machine (Queens, São Paulo, BRA) with the knee and hip at 90° of flexion and the ankle in a neutral position (figure 2). For the MVIC hip abduction (0° and 30°) tests, the subjects were positioned on their sides lying on a divan with the test lower limb placed above and with both lower limbs positioned at neutral hip and knee flexions, as measured by the investigator. For the maintenance of these positions the thighs were stabilized with padding and the calves were fixed with a belt applied immediately distal to the knees (Figure 3 and 4).

Before data collection procedures began, each subject received a verbal explanation and a demonstration of the testing activities and practice trials were performed to ensure the subject's comprehension and safety. After familiarization, the subjects randomly performed three MVIC for 5 seconds with a 2 minute rest between repetitions and 10 minutes between each set of exercises to prevent muscle exhaustion. EMG signals were collected throughout each MVIC and verbal encouragement was provided throughout the testing.

### Data analysis

The normalization of the VMO, VLL and VLO EMG signals for the three exercises (knee extension at 90° of flexion, MVIC hip abduction at 0° of abduction, MVIC hip abduction at 30° of abduction) were obtained dividing the highest EMG value of the MVIC trials by the EMG value of a MVIC knee extension at 50° of flexion in a seated position and multiplied by 100 [16]. The normalization procedure performed at 50° of knee flexion in a seated position followed the same protocol of positioning for the test at 90° of knee flexion in a seated position. Normalized EMG readings were analyzed by two-way analysis of variance with repeated measurements. *Post hoc *comparisons within the values obtained for each of the muscles were realized by the Bonferroni multiple comparisons analysis. Significance was defined as p ≤ 0.05.

## Results

**Figure 5:**
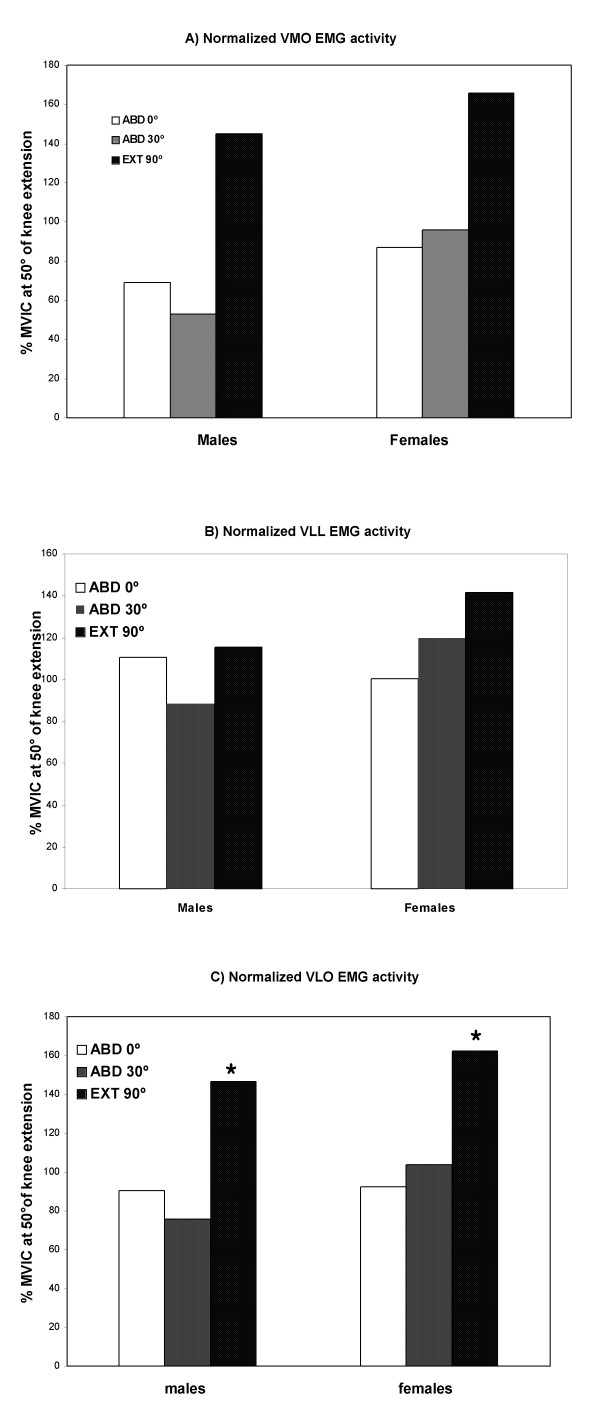
Normalized EMG activity expressed as RMS values from healthy subjects performing MVIC knee extension at 90° of flexion, MVIC hip abduction at 0° and 30° of abduction. **A) **Significantly higher VMO activity for both male and female subjects performing MVIC knee extension at 90° of flexion when compared to MVIC hip abduction at 0° and 30° of abduction. **B) **VLL EMG activity without significant differences among the three exercises tested. **C) **Significantly higher VLO activity for male subjects was observed in MVIC knee extension at 90° of flexion compared with MVIC hip abduction at 0° and 30° of abduction and for female subjects performing MVIC knee extension at 90° of flexion when compared with MVIC hip abduction at 0° and 30° of abduction.

Maximum voluntary isometric contractions for knee extension at 90° of flexion resulted in a significantly higher EMG activity for the VMO muscle compared with hip abduction at 0° and 30° of abduction for both male (p < 0.008) and female (p < 0.0005) subjects (Table 1, Figure 5).

**Table 1:** Summarized results of normalized EMG values of VMO, VLL and VLO muscles during MVIC knee extension at 90° of flexion and MVIC hip abduction at 0° and 30° of abduction (N = 21) The results are shown as a percentage of MVIC at 50° of knee flexion in a seated position

Test procedure	VMO (%)	VLL (%)	VLO (%)
Hip abduction at 30°			
Male	53.70	88.31	75.70
Female	96.07	119.61	103.98

Hip abduction at 0°			
Male	69.05	110.62	90.25
female	87.09	100.13	92.30

MIVC at 90° of knee flexion			
Male	144.91 ^c^	115.31	146.72 ^d^
female	165.97 ^b^	141.30	162.61 ^a^
p ≤ 0,05			

Letters demonstrate higher EMG activity of the same muscle in which there were statistically significantly differences between the three exercises

^a ^p = 0,02196 High EMG activity MVIC knee extension at 90° of flexion **vs **MVIC hip abduction at 0° of abduction

^b ^p = 0,008 High EMG activity MVIC knee extension at 90° of flexion **vs **MVIC hip abduction at 0° and 30° of abduction

^c ^p = 0,000055 High EMG activity MVIC knee extension at 90° of flexion **vs **MVIC hip abduction at 0° and 30° of abduction

^d ^p = 0,000761 High EMG activity MVIC knee extension at 90° of flexion **vs **MVIC hip abduction at 0° and 30° of abduction

There were no statistically significant differences in the VLL EMG activity among the three types of exercise tested (Table 1, Figure 5). The VLO muscle demonstrated a similar pattern to the VMO muscle showing higher EMG activity in MVIC knee extension at 90° of flexion compared with MVIC hip abduction at 0° and 30° of abduction for male (p < 0.0007) and with MVIC hip abduction at 0° of abduction for female subjects (p < 0.02196) (Table 1, Figure 5). There were no significant differences between gender with respect to the EMG activity of the VMO, VLL and VLO muscles among the three types of exercise tested.

No selective EMG activation was observed when comparison was made between the VMO, VLL and VLO muscles while performing MVIC knee extension at 90° of flexion and MVIC hip abduction at 0° and 30° of abduction for both male and female subjects.

## Discussion

The primary purpose of this article was to investigate whether VMO, VLL and VLO EMG activity can be influenced by hip abduction. The results showed that no selective EMG activation was observed when comparison was made between the VMO, VLL and VLO muscles while performing MVIC knee extension at 90° of flexion or MVIC hip abduction at 0° and 30° of abduction for both male and female subjects. These results are in agreement with Hertel et al. [4] who investigated the EMG activity of the VMO, VLL and gluteus medius in eight healthy young adult volunteers with no history of knee injury while performing uniplanar knee extension, knee extension/hip adduction, knee extension/hip abduction and found no significant differences in the VMO/VL ratio between the exercises. It is well established in the literature that the isolation of any of the quadriceps components or selective strengthening is unlikely, especially concerning the VMO/VLL muscles [28,29]. Our findings demonstrate that hip abduction do not facilitated VLL and VLO activity in relation to the VMO in healthy subjects performing maximum voluntary isometric contraction.

We are unaware of any studies which have demonstrated the characteristics of EMG activity between VMO, VLL and VLO in subjects performing hip abduction in different positions.

The secondary purpose of the present work was investigating the EMG activity of the VMO, VLL and VLO while performing the three exercises tested. The results showed that the VLO presents a significantly different behavior compared with the VLL, whereas the VLO showed a similar motor unit recruitment to the VMO, producing higher EMG activity in MVIC knee extension at 90° of flexion compared with MVIC hip abduction at 0° and 30° of abduction. No significant differences were found in the EMG activity of the VLL among the three exercises tested, thus verifying that the VLL and VLO demonstrate not only anatomical but distinct motor unit recruitment characteristics. Bevilaqua-Grossi et al. [27] investigated the EMG activity of the VMO, VLL and VLO muscles in 21 healthy subjects performing open kinetic chain knee extension at 15° and 90° of flexion. The results showed that the VLO and VMO were more active at 90° of flexion compared with the higher activity of the VLL at 15° of flexion, demonstrating that the VMO and VLO muscle have the same behavior suggesting a synchronic antagonist stability role of the patella in healthy people. The striking difference between VLL and VLO behavior concerning the EMG activity observed, could be because the VLL fiber alignment tends to traction the patella, offering greater contribution to knee extension than patella stabilization, different from the VLO which spirally and inclination fibers in relation to femoral dyaphisis promotes patella alignment associated with the VMO [24]

Hertel et al. [4] reported that both the VMO and VL are more activated in uniplanar knee extension when compared with knee extension/hip adduction or abduction. These results are not entirely supported by our results, in which the VLL showed no significant differences between MVIC knee extension at 90° of flexion compared with MVIC hip abduction at 0° and 30°. These conflicting results concerning the VLL recruitment pattern may be because these authors tested the exercises in closed kinetic chain while the three exercises tested in the current work were performed in open kinetic chain.

Possible limitations of this study may be related to the non collection of gluteus medius EMG activity during the three exercises tested. Normalization methods using other types of muscle contractions or other angles of the knee joint may result in different VMO/VLL/VLO ratios between studies that use different methods of normalization. The absence of increased EMG activity with hip abduction could have been due limitations in EMG recordings from MVCs. Results might be different when studying submax voluntary contractions. Although normalized EMG data are useful in measuring relative levels of activity between muscles, such information is not indicative of muscular strength or muscular balance [58].

Future studies of this research group will focus on subjects with patellofemoral pain and investigate the recruitment patterns provided from such subjects performing the same task.

### Clinical implications

Bevilaqua-Grossi et al. [24] dissected the thighs of 32 human cadavers and determined the anatomical organizations of the VLL and VLO muscles. The distal fibers of the VLO were interdigitated with the lateral retinaculum and the iliotibial tract in all specimens, which subsequently joined in a common tendon with the VLL on the superolateral border of the patella. These results agree with previous anatomical studies which describe the origin [30-34] and insertion of these muscles [35,36]. Thus, tightness of the lateral retinaculum, perhaps as a result of increased tension in the iliotibial tract, could potentially alter the tracking of the patella in the trochlear groove, becoming an important factor in the etiology of patellofemoral pain [37]. Following this theory, some clinicians contraindicate rehabilitation exercises using hip abduction in patients with patellofemoral complaints based on the premise of avoiding excessive tightness of the lateral structures or a VLL and VMO imbalance, since the anatomical origin of the iliotibial tract has a close relation with the iliotibial band and the gluteus medius muscles [21,38]. This theory is not supported by recent works which investigated the role of pelvic control as a contributing factor in the development of anterior knee pain [39-42].

Ireland et al. [39], using hand held dynamometers, investigated hip abduction and external rotation isometric strength in 15 female patients with patellofemoral pain compared with a control group. They found significant weakness of the hip abductors and external rotators of the patellofemoral pain group. It is postulated that in the absence of pelvic control due to hip abductor and external rotator weakness, the femur may adduct and internally rotate, further increasing lateral patellar contact pressure [43]. These findings suggest that hip abduction exercises may be indicated and necessary for patients with patellofemoral pain who present absence of satisfactory pelvic control.

## Conclusion

The results showed that no selective EMG activation was observed when comparison was made between the VMO, VLL and VLO muscles while performing MVIC at 30° and 0° of hip abduction and 90° of knee flexion for both male and female subjects. Our findings demonstrate that hip abduction do not facilitated VLL and VLO activity in relation to the VMO, however, this study included only healthy subjects performing maximum voluntary isometric contraction contractions, therefore much remains to be discovered by future research
